# Management of COVID-19-Associated Guillain-Barré Syndrome in a Full-Term Pregnant Woman: A Case Report

**DOI:** 10.34763/jmotherandchild.20232701.d-22-00066

**Published:** 2023-08-07

**Authors:** Anouar Jarraya, Manel Kammoun, Sonda Dammak, Kamel Kolsi

**Affiliations:** Department of Anesthesiology and Intensive Care, Hedi Chaker University Hospital, University of Sfax, Sfax, Tunisia

**Keywords:** Plasmapheresis, Guillain-Barré syndrome, COVID-19, Pregnancy, Case report

## Abstract

Guillain-Barré syndrome (GBS) can occur after viral infections. Its occurrence after COVID-19 infection in the peripartum period is a very rare co-occurrence. Therefore, there are no guidelines for the management of these patients. We report the case of a 32-year-old pregnant woman who developed COVID-19-associated GBS with aspiration pneumonia, motor weakness, and ascending paralysis at 39 weeks of gestation. Preoperative plasmatic exchange (plasmapheresis) and oxygen support were very effective and allowed for a rapid recovery within five days. Because of foetal distress during labor, the patient had a caesarean section under spinal anaesthesia with no maternal complications or adverse foetal outcomes.

## Introduction

Guillain-Barré syndrome (GBS) is a rare, acute, auto-immune-mediated polyradiculoneuropathy that can start a few days or weeks following a viral infection [1]. A few cases of COVID-19-associated GBS in the general population [1] and during pregnancy [2] have been reported. Its co-occurrence during a full-term pregnancy is uncommon and can result in significant maternal and perinatal morbidity [2]. The obstetrical and anaesthesia management of this risky situation is not yet well known, so there are no guidelines available [3]. In this case report, we describe our experience of successful and original preoperative management allowing for the improvement of GBS clinical features before a non-complicated caesarean delivery under spinal anesthesia.

## Case presentation

*Patient Information:* A 32-year-old, gravida 3, para 3 woman with no previous comorbidities was admitted for respiratory distress at 39 weeks’ gestation. She had experienced a mild form of COVID-19 during the Omicron wave with arthralgia, myalgia, a 38°C fever, and a runny nose seven days earlier. Our patient was unvaccinated.

*Clinical Findings:* At admission, the physical examination showed hypoxemia with 88% oxygen saturation. Routine laboratory screening tests and full blood analysis were all within normal limits (haemoglobin: 11.3 g/dL; platelet: 202 ×10^6^/mm^3^; WBC: 10.2× 10^9^; prothrombin ratio: 98%; fibrinogen concentration: 6.1 g/L; creatinin: 65μmol/L; albumin: 43 g/L; no cytolysis, and no ionic disorders). A COVID-19 screening with reverse transcriptase–polymerase chain reaction tests (nasolaryngeal swab) had been positive seven days earlier. A chest X-ray showed aspiration pneumonia and a transthoracic echocardiography showed 77% ejection fraction without any abnormalities. Continuous foetal heart rate monitoring showed the absence of foetal distress. An ultrasound of the abdomen was completed for assessment of foetal well-being, and showed a live fetus, 39 weeks of gestation (WG), appropriate for gestational age.

*Timeline of current episode:* The next day, the patient had progressively ascending, bilaterally symmetric muscle weakness. The deep tendon reflexes were reduced. The patient had a rapid onset of dysphagia, urinary incontinence, and incapacity to walk but without sensory deficit or disturbance of any cranial nerve function.

*Diagnostic assessment:* The patient was referred to the intensive care unit with a presumed diagnosis of Guillain-Barré syndrome. Cerebral MRI imaging was normal. An electromyography was completed and showed a severe demyelinating motor-sensory neuropathy, which is compatible with the diagnosis of GBS.

*Diagnosis:* The final diagnosis was COVID-19-associated Guillain-Barré syndrome in a full-term pregnant woman.

*Therapeutic interventions:* The pulmonary aspiration was treated by oxygen supplementation (progressive oxygen supplementation from 8L/min at the beginning to CPAP at the peak of neurological signs), antibiotherapy (amoxicillin clavulanic acid), fluid replacement with furosemide, and paracetamol under continuous monitoring of the baby. Our patient had spaced uterine contractions with cervical effacement. As such, a tocolytic treatment was prescribed (phloroglucinol and nicardipine) to delay delivery after the plasmapheresis and after improving respiratory conditions. The patient had three sessions of plasmapheresis within the following five days.

*Follow-up and outcome of interventions:* The evolution of our patient's status was favorable. A clinical improvement in the neurological status was observed four days after the first plasmapheresis. We noted a total recovery regarding dysphagia, areflexia, and muscle weakness. Our patient was able to walk without any assistance, and she showed no motor weakness in the upper or lower extremities. Her respiratory conditions improved, and the need for oxygen supplementation was reduced to 2 L/min with the nasal cannula. However, blood analysis showed reduced albumin concentration (24 g/L), reduced fibrinogenemia (2.6 g/L), correct hemostatic status (prothrombin ratio: 79%), and no ionic or metabolic disorders. The patient then escaped the tocolysis and presented with acute foetal distress, and an emergent caesarean section was indicated. The patient had spinal anaesthesia with subarachnoid 10 mg bupivacaine, 2.5 μg sufentanyl, and 100 μg morphine. No adverse or unanticipated maternal or foetal events were noted. Her postpartum course was uncomplicated, with no relapse of symptoms. Her baby, a 3040-gram male newborn, was healthy, had a 9/10 Apgar score at birth, and was breastfed when the mother was discharged. We continued the same treatment postoperatively, and she had 2 other sessions of plasmapheresis in postpartum with an enhanced recovery strategy. The patient was safely discharged home on the fifth postoperative day.

*Patient Perspective:* During the hospital stay and at discharge, the patient was very satisfied with the quality of healthcare.

*Informed Consent and ethics committee approval:* Approval from the local ethics committee and informed consent to publication were obtained before the publication of this case report.

## Discussion

Guillain-Barré syndrome, an inflammatory auto-immune polyradiculoneuropathy associated with numerous viral infections, may occur after the SARS-CoV2 viral process [4]. Some cases were reported after COVID-19 vaccination [5], while other studies showed that the prevalence of SARS-CoV2-associated GBS has declined since the introduction of vaccines [6]. In pregnant women, COVID-19-associated Guillain-Barré syndrome can occur at any time during gestation with atypical clinical features and difficult diagnosis [7] and can lead to adverse maternal and perinatal outcomes [8]. Even if the severity of COVID-19 in the Omicron wave was reduced [9], we think that the risk of serious complications like GBS is always present, especially in unvaccinated patients [10].

The choice of an anaesthesia technique was difficult given the conditions of our patient. Spinal anaesthesia may have some limits in patients with motor weakness in the lower extremities. Nevertheless, general anaesthesia for a full-stomach pregnant patient with pneumonia and dysphagia seems to be associated with a high risk of difficult intubation, aspiration, and cardiac arrest caused by GBS-related dysautonomia or the use of succinylcholine, which was implicated in a cardiac arrest with severe hyperkalemia after a crush induction in a pregnant woman recovering from GBS [11]. As a result, opting for the treatment of GBS and its complications, like pneumonia, before delivery was beneficial. On the other hand, general anaesthesia may worsen foetal outcomes, particularly in cases of acute foetal distress and foetalbradycardia. Previous studies showed poor outcomes in neonates whose mothers suffered from GBS [8]. There were high rates of pre-term birth (35.7%) and stillbirth (8.33%) with a 66.7% rate of spontaneous vaginal delivery [8].

In our case, plasmapheresis was safe and effective during pregnancy, but it required continuous maternal and foetal monitoring. We should mention that special hygiene measures are mandatory to prevent nosocomial infections for pregnant women with GBS, who are vulnerable to viral and bacterial infections, and this risk may increase with plasmapheresis. Daily blood samples to control the hemostatic status and plasmatic protein concentrations like albumin, prothrombin, and particularly fibrinogen are needed. These blood samples are mandatory because these factors can decrease after plasmapheresis. Finally, we recommend an enhanced recovery strategy after cesarean section delivery for these patients, in order to reduce postpartum complications [12].

### Key messages

- COVID-19-associated GBS in pregnancy is a rare co-occurrence.
- GBS in pregnancy can cause adverse maternal and perinatal outcomes.
- Anaesthesia for pregnant women with GBS is a risky situation.
- Clinical features of GBS may be improved by plasmapheresis which may reduce the risks related to anesthesia.
- Plasmaphereis for pregnant women with GBS requires special hygiene measures and continuous monitoring.
